# Confidence in Emotion Perception in Point-Light Displays Varies with the Ability to Perceive Own Emotions

**DOI:** 10.1371/journal.pone.0042169

**Published:** 2012-08-22

**Authors:** Britta Lorey, Morten Kaletsch, Sebastian Pilgramm, Matthias Bischoff, Stefan Kindermann, Isabell Sauerbier, Rudolf Stark, Karen Zentgraf, Jörn Munzert

**Affiliations:** 1 Institute for Sports Science, Justus Liebig University Giessen, Giessen, Germany; 2 Bender Institute of Neuroimaging, Justus Liebig University Giessen, Giessen, Germany; 3 Institute for Sports Science, University of Munster, Munster, Germany; 4 Cognitive Neuroscience Group Center for Psychiatry and Psychotherapy, Justus Liebig University Giessen, Giessen, Germany; Katholieke Universiteit Leuven, Belgium

## Abstract

One central issue in social cognitive neuroscience is that perceiving emotions in others relates to activating the same emotion in oneself. In this study we sought to examine how the ability to perceive own emotions assessed with the Toronto Alexithymia Scale related to both the ability to perceive emotions depicted in point-light displays and the confidence in these perceptions. Participants observed video scenes of human interactions, rated the depicted valence, and judged their confidence in this rating. Results showed that people with higher alexithymia scores were significantly less confident about their decisions, but did not differ from people with lower alexithymia scores in the valence of their ratings. Furthermore, no modulating effect of social context on the effect of higher alexithymia scores was found. It is concluded that the used stimuli are fit to investigate the kinematic aspect of emotion perception and possibly separate people with high and low alexithymia scores via confidence differences. However, a general difference in emotion perception was not detected in the present setting.

## Introduction

One striking characteristic of human nature is our ability to observe, recognize, and evaluate the emotions of our conspecifics. In earlier times, this represented a clear evolutionary advantage for the acting individual. Today, disregarding the emotional constitution of a person rarely results in a threat to life, but repeated disregard still leads to confusion, uncertainty, terminated relationships, and—at worst—social isolation. Hence, it is still crucial for us to detect and evaluate the emotional signals given by our conspecifics' appearance and behavior in order to avoid adverse consequences. It could even be argued that a deficit in emotion perception may lead to negative effects on social relationships that could relate in turn to psychiatric disorders and somatic diseases [1]–[3].

One fundamental issue in this context is whether and to what extent the perception of the emotions of others depends on the ability to perceive, recognize, and evaluate one's own feelings and emotions. The idea that perceiving emotions in others relates to activating the same emotion in oneself has been the topic of several theoretical papers and some experimental studies [4]–[6]. For example, it has been reasoned that people with the personality trait alexithymia, who have shortcomings in or are unable to perceive, recognize, and describe their emotions, are also impaired in the perception and recognition of verbal and nonverbal emotional stimuli [7] and in the recognition of emotions from facial expressions [8]. Neurobiological findings have demonstrated that persons with high alexithymia scores reveal less neural activation in emotion-processing structures such as the anterior cingulate and medio-frontal areas of the brain while observing emotional stimuli compared to persons with low alexithymia scores. Thus, a differential ability to recognize emotions seems to relate to a differential activation of brain regions representing own emotion processing [9].

Most available alexithymia studies have used human facial expressions to examine how observers are able to perceive and recognize another person's emotional state. However, research has shown that not just the face but the whole body has the potential to express emotional states [10]–[12]. Since the seminal work of Johansson in 1973 [13], it is known that human actions can be perceived intuitively even when the only information available to an observer comes from just a few points representing the joints of the body. Such research is implemented experimentally with the so-called point-light technique. This records the kinematics of a few dots placed on a model's body, and uses these to reconstruct point-light displays (PLDs). PLDs have been applied to study not only gait direction or gender recognition [14], [15] but also how human movements represent an individual's emotional state. Such research has revealed that emotions can be detected reliably even when facial expression is not visible, and emotion perception and recognition can draw only on the biological movement and its kinematics [16]. The advantage of using highly simplified PLDs is that they provide only kinematic movement information, thereby ensuring that the recognition process is not influenced by confounding variables in the stimulus material found in complex and natural stimuli such as facial information or stimulus intensity [17]. Thus, task demands are specified clearly (using kinematic information to detect emotions) and the available information can be controlled.

Recently, attention has been directed toward possible factors that modulate the detection of emotion in PLDs. For example, Clarke et al. [11] investigated the influence of social context information on emotion perception by depicting PLDs with either one person or two persons in an interpersonal emotional dialogue. They demonstrated that the contextual social information of interacting persons enhances the perception of the emotion quality. Research has also addressed interindividual differences. For example, Alaerts et al. [18] investigated potential gender differences in a series of tasks involving the general recognition of biological motion and the recognition of the emotional state of PLD figures and found some gender-dependent differences in recognizing emotions depicted by PLDs. Moreover, several studies have demonstrated that neuropsychiatric disorders such as autism also influence the ability to recognize emotions depicted in PLDs [19], [20]. Both adults and children with an autism spectrum disorder were significantly less able to recognize emotional displays. However, they had no difficulties in recognizing nonemotional displays in a control task, suggesting that they are impaired in attending to emotional states.

It has been suggested that one possible underlying mechanism mediating the perception and recognition of emotions in human movements is based on embodied simulation. Seeing someone else's emotional expression might be linked to a simulation of the respective emotion in oneself and, therefore, to experiencing one's own emotions [4], [21]. The most prominent neurophysiological finding in support of such embodied simulation processes was the discovery of so-called mirror neurons within premotor and parietal areas of the brain that fire during action observation processes (see, for a review, [22]). The functional role of these mirror neurons has been a topic of major debate among neuroscientists, psychologists, and philosophers. Interpretations of activity in the mirror-neuron system range from facilitation of imitative behavior [23], across action understanding [24], language development [25], and the implementation of perception–action circuits from a common-coding perspective [26], up to the aforementioned emotional simulation processes [4], [18], [21], [27]. Mirror neuron activity, therefore, is hypothesized to be the neural mechanism by which observed movements are matched onto the observer's own body representations in order to understand the actions, intentions, or emotions of the individual being observed. Accordingly, understanding emotions, like understanding actions, is related to a process of simulating the observed emotions that is based on the observer's representations [4], [12], [21].

Against this background, we hypothesized that people who reveal a trait close to alexithymia would demonstrate difficulties in perceiving emotions depicted by kinematic movement stimuli. Therefore, we used PLDs of human actions of varying difficulty to investigate the effect of the personality trait alexithymia on emotion perception. More precisely, we tried to elucidate the effects of low and high alexithymia scores on (a) the perception and evaluation of emotional states depicted by PLDs, (b) the subjective confidence in this evaluation, and (c) the modulating effects of the social context (monades versus dyades). We hypothesized that alexithymia scores would influence each of the topics under examination.

## Materials and Methods

### 2.1. Ethical Statement

The study was specifically approved by the local ethics committee (local ethics commission, Department of Psychology and Sports Science, University of Giessen), and all participants gave their informed written consent in accordance with the Declaration of Helsinki.

### 2.2. Participants

A total of 95 participants (48 female, mean age = 41.5 years, *SD* = 13.5) with normal or corrected-to-normal vision completed the study. Age as well as level of education were balanced within the sample. None of the participants reported any history of psychiatric or neurological disorders and no history or current use of any psychoactive medication.

Their alexithymia score was assessed with the German version of the Toronto Alexithymia Scale (TAS-26, [28]). The TAS-26 is a 20-item instrument that is commonly used to assess alexithymia. Items are rated on a 5-point Likert scale ranging from 1 (*strongly disagree*) to 5 (*strongly agree*). The TAS-26 has three subscales. The first subscale measures the difficulty in describing own feelings and emotions (five items). The second subscale is used to measure the difficulty in identifying emotions (seven items). The third subscale identifies the tendency of individuals to focus their attention externally (eight items). The average scores of the present participants ranged from 1.24 to 3.24 (*M* = 2.15; *SD* = 0.42) on a scale from 1 (*low TAS score*) to 5 (*high TAS score*).

### 2.3. Creating Point-Light Displays

Seven pairs of two actors provided the movements for the point-light displays (PLDs). Each pair was asked to perform an interaction portraying one of four emotional scenes: anger, sadness, joy, and love. Scenes with anger and sadness were pooled in the category “negative,” and scenes with love and joy were pooled in the category “positive.” Prior to acting, both actors were given a script instructing them to perform the same emotion in order to produce a behavioral pattern that was as symmetrical as possible. Actors were asked to act out the emotion immediately. They were completely free to express their emotions in whatever way they liked—for example, by overt symbolic gestures. At least four clips of each pair and each emotional scene were produced. In addition, for each of the dyadic PLDs (scene with two actors: dyade), a monadic PLD version was created that consisted of the dots of one of the two individuals alone (scene with one actor: monade). Apart from this, they still displayed the same emotion with the same movements. This resulted in a corpus of about 96 recordings with 8 recordings for each category (Monade vs. Dyade×Positive vs. Negative×3 Difficulty Levels, see next section below).

**Figure 1:**
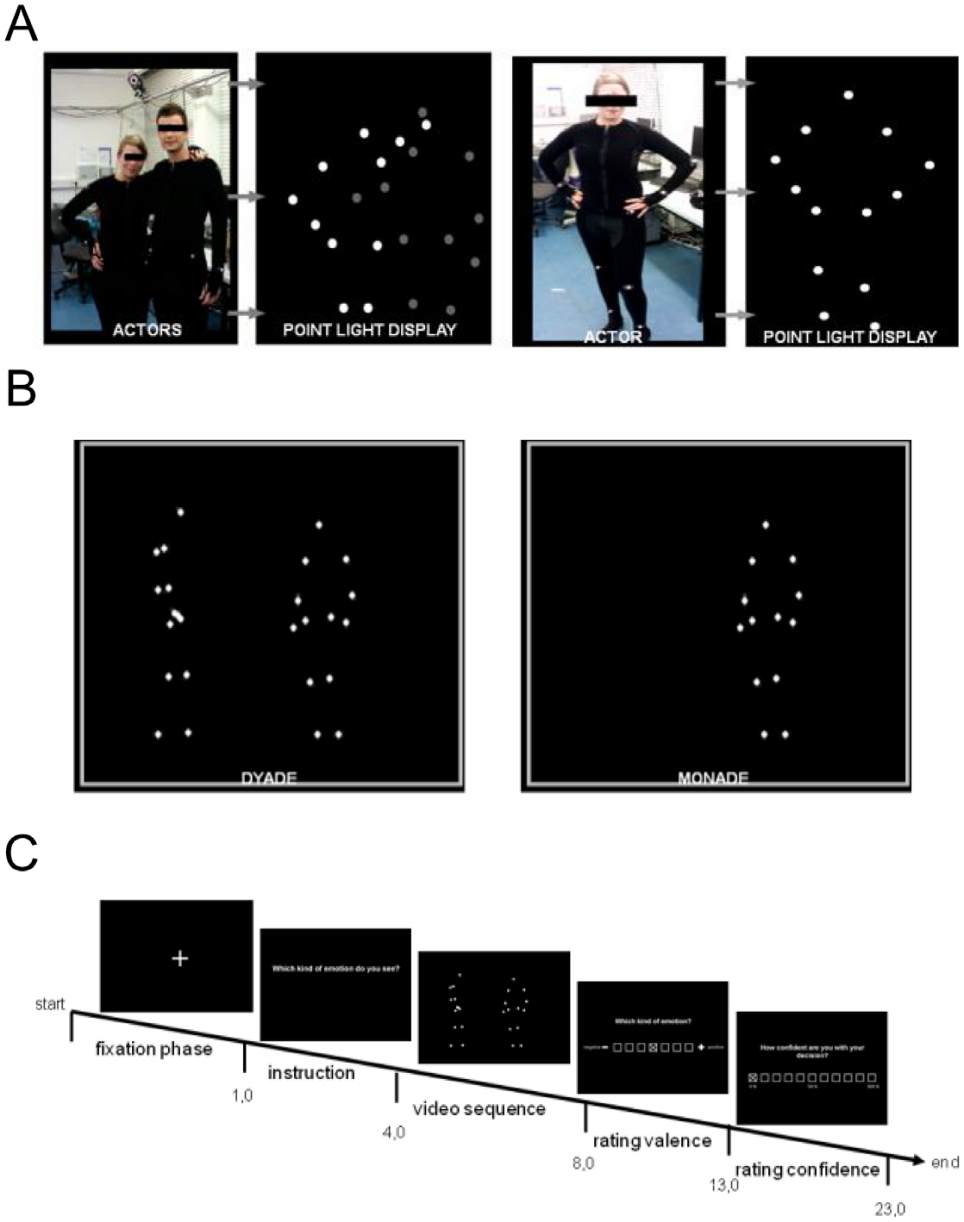
Preparation of stimuli. To create the point-light displays, 13 reflective markers were attached to an actor's head, shoulders, elbows, wrists, hips, knees, and ankles. They were then tracked using a Vicon motion-capture system. (A) Photograph of actors with 13 markers attached to the body and the corresponding point-light figures. (B) Examples of dyadic and monadic point-light displays. (C) Temporal structure of the experiment.

All scenes were recorded with a 12-camera VICON MX system (Oxford Metrics, Oxford, England) operating at 100 Hz. Thirteen reflective markers were attached to defined anatomical landmarks on the upper body (including the shoulders, the elbow joints, the wrists, and the forehead) and the lower body (including the hips, the knee joints, and the ankles) of each actor (Figure 1). After capturing, data postprocessing was conducted with Nexus 1.5.2 (Vicon Motion Systems, Oxford, England) in order to calculate 3-D coordinates of the markers. The video files were created in a two-step process using Matlab software (MathWorks, Natick, MA). First, for each timepoint, the 3-D coordinates of the 13 markers were plotted as white spheres on a black background. Then, the frames of the captured scenes were rendered as audio-video interleaved (avi) movie files at a frame rate of 25 Hz. For each scene, video files with a duration of 4 s were created from a front view. In all presented PLDs, the dots appeared white against a black background at an approximate viewing distance of 50 cm.

### 2.4. Stimuli: Validation and Determination of Difficulty

Prior to the experiment, an index of difficulty was determined for all recorded PLDs in order to separate the recordings into three classes (easy, medium, and difficult to recognize). We asked 30 participants who did not participate in the present experiment to evaluate the negativity or the positivity of the emotions displayed in the videos in a forced-choice paradigm. The three categories of difficulty were created by calculating the percentage of people who agreed on the valence of the video scene. Thus, easy videos were defined by a consensus of 91–100%; medium videos, by a consensus of 71–90%; and difficult videos, by a consensus of 50–70%.

### 2.5. Procedure

Prior to or after the actual experiment, participants attended a control session, so that experimenters could assess data ensuring that all participants were able to recognize movements from PLDs. They were given control stimuli depicting sports movements such as juggling and basketball, and asked to give a brief definition of each movement as quickly as possible. One-half of the participants started with experiment and the other half with the control session in order to control for sequence effects.

The experiment presented a series of 96 video trials (8 sequences per condition: Monades vs. Dyades×Negative vs. Positive Emotions×3 Difficulty Levels). Conditions were presented in a pseudo-randomized order counterbalanced across participants. Each trial started with a fixation phase (1 s), followed by the instruction (3 s) and the respective video sequence (4 s). After observing this sequence, participants were asked to assess the emotional valence of the videos on a 7-point scale ranging from 1 (*negative*) to 7 (*positive*) with 4 marking the neutral center of the scale. The position of the valence label (*negative*) was altered from the left to the right side for one-half of the participants. After each valence rating, participants were asked to report how confident they were about their rating on an 11-point scale ranging from 1 (*0% confidence*) to 11 (*100% confidence*).

### 2.6. Data Analysis and Statistics

Because of the different scale orientation in negative videos, we first repoled the participants' scaling so that it was the same for both types of videos (most positive scored = 7, least positive scored = 1; most negative scored = 7, least negative scored = 1). All further analyses were performed with the repoled data.

We calculated mean scores for each rating and each experimental condition. To explore the potential differences between participants with high versus low alexithymia and to understand the effect of own emotions on both evaluating emotions and the confidence of emotion perception, we computed a repeated measures ANOVA for each rating in order to examine the effects of the valence of interaction (positive vs. negative), the social context (monades vs. dyades), the difficulty of videos (easy, medium, difficult), and alexithymia as a categorical between-group factor. To create the between-group factor, we generated extreme groups by taking the 25% with the highest (*n* = 24, *M* = 1.61, *SD* = 0.17) and the 25% with the lowest TAS-26 scores (*n* = 24, *M* = 2.68, *SD* = 0.19). The two groups differed significantly with respect to their TAS-26 scores, *t*(46) = −20.47, *p*<.001.

Additionally, we correlated the two rating scores with the TAS-26 score for all participants and calculated a correlational analysis over the whole group.

All statistics were calculated using SPSS software (Version 19), and an alpha level of .05 was used for all statistical tests.

## Results

### 3.1. Control Data

#### 3.1.1. Control session: biological motion recognition test

Participants were able to identify each of the actions reliably and far above chance level. On average, 92.3% (range: 66.7%–100%) of the classifications were correct. One participant with 60% incorrect classifications was excluded from the study. The two groups completing the control session either before or after the main experimental session did not differ in their ratings of either emotional valence, *F*(1, 95)<1, *ns*, **or** confidence, *F*(1, 95)<1, *ns*.

#### 3.1.2. Position of valence label during valence rating

The groups with the valence label “negative” on the left versus the right side during the valence rating did not produce systematically different valence ratings, *F*(1, 96)<1, *ns*, or confidence ratings, *F*(1, 96)<1, *ns*, in the main trail.

### 3.2. Influence of Alexithymia on Valence and Confidence Rating

A 2 (Valence)×2 (Social Context)×3 (Difficulty) repeated measures ANOVA with alexithymia as a categorical between-group factor was computed for the valence and the confidence rating (Table 1). Since we were mainly interested in the effect of alexithymia, we will first focus on main effects and interactions containing this factor. Results mainly driven by the additional factors valence, social context and difficulty are presented in the next paragraph.

**Table 1:** Statistical Data of Valence×Social Context×Difficulty Repeated-measures ANOVA for Valence and Confidence Rating.

Rating of Emotional Valence	*df*	*F*	*η^2^*	*p*
Alexithymia (between-group factor)	1,46	.27	.01	.605
Valence	1,46	.90	.02	.347
Valence*Alexithymia	1,46	.25	.01	.613
Social Context	1,46	383.75	.89	.001*
Social Context*Alexithymia	1,46	1.77	.08	.190
Difficulty	2,92	165.99	.78	.001*
Difficulty*Alexithymia	2,92	3.20	.07	.045
Valence*Social Context	1,46	.33	.01	.57
Valence*Social Context*Alexithymia	1,46	.39	.01	.54
Valence*Difficulty	2,92	11.89	.21	.001*
Valence*Difficulty*Alexithymia	2,92	.28	.01	.75
Social Context*Difficulty	2,92	6.12	.12	.003*
Social Context*Difficulty*Alexithymia	2,92	.01	.00	.99
Valence*Social Context*Difficulty	2,92	39.11	.46	.001*
Valence*Social Context*Difficulty*Alexithymia	2,92	.34	.01	.71

Confidence Rating	*df*	*F*	*η*	*p*
Alexithymia (between-group factor)	1,46	4.13	.08	.048*
Valence	1,46	10.89	.19	.002*
Valence*Alexithymia	1,46	2.07	.04	.16
Social Context	1,46	125.78	.73	.001*
Social Context*Alexithymia	1,46	.59	.01	.45
Difficulty	2.92	84.77	.65	.001*
Difficulty*Alexithymia	2.92	.67	.01	.51
Valence*Social Context	1,46	10.56	.18	.003*
Valence*Social Context*Alexithymia	1,46	.00	.00	.98
Valence*Difficulty	2.92	3.75	.09	.026*
Valence*Difficulty*Alexithymia	2.92	1.08	.02	.35
Social Context*Difficulty	2.92	9.89	.18	.001*
Social Context*Difficulty*Alexithymia	2.92	.18	.00	.83
Valence*Social Context*Difficulty	2.92	15.99	.26	.001*
Valence*Social Context*Difficulty*Alexithymia	2.92	.55	.01	.58

*Note.* ANOVA = Analysis of variance.

* *p*<.05.

**Figure 2:**
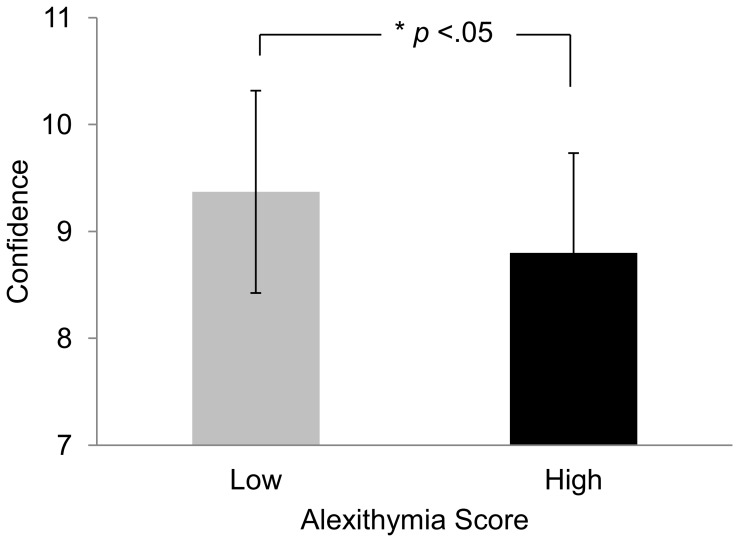
Differences in confidence of people with high and low alexithymia scores. Average confidence ratings and their standard deviations are displayed as a function of participant group (high and low alexithymia). The difference is significant at the .05 level.

**Figure 3:**
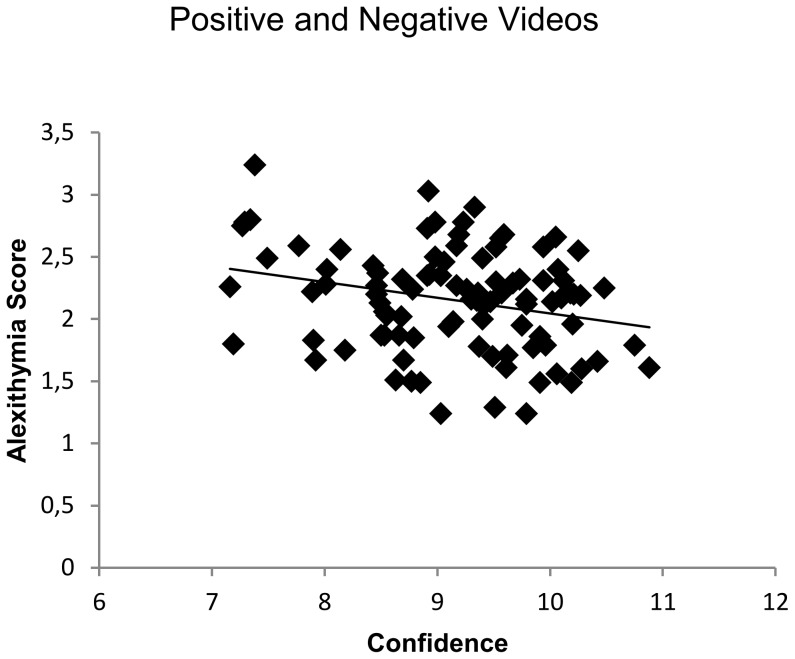
Rating confidence correlates with the alexithymia score. Linear fit for rating confidence and alexithymia (negative and positive videos).

For the confidence rating, the analyses revealed a significant effect of alexithymia on confidence, *F*(1, 46) = 4.42, *p*<.05, η^2^ = 0.08. Participants with low alexithymia scores assessed the depicted videos more confidently than participants with high alexithymia scores (Figure 2). Additional correlational analyses of the confidence rating with the alexithymia scores of all participants revealed a significant correlation for the confidence rating, *r* = −.26, *p* = <.01, *R* ^2^ = .069 (Figure 3).

For the valence rating, the two-way interaction between difficulty and alexithymia was also significant, *F*(1, 46) = 3.204, *p*<.05, η^2^ = 0.07: Differences between easy, medium, and difficult videos were smaller for people with high TAS-26 scores compared to people with low TAS-26 scores.

### 3.3. Additional influence of Valence, Social Context and Difficulty on Valence Rating and Confidence Rating

**Figure 4:**
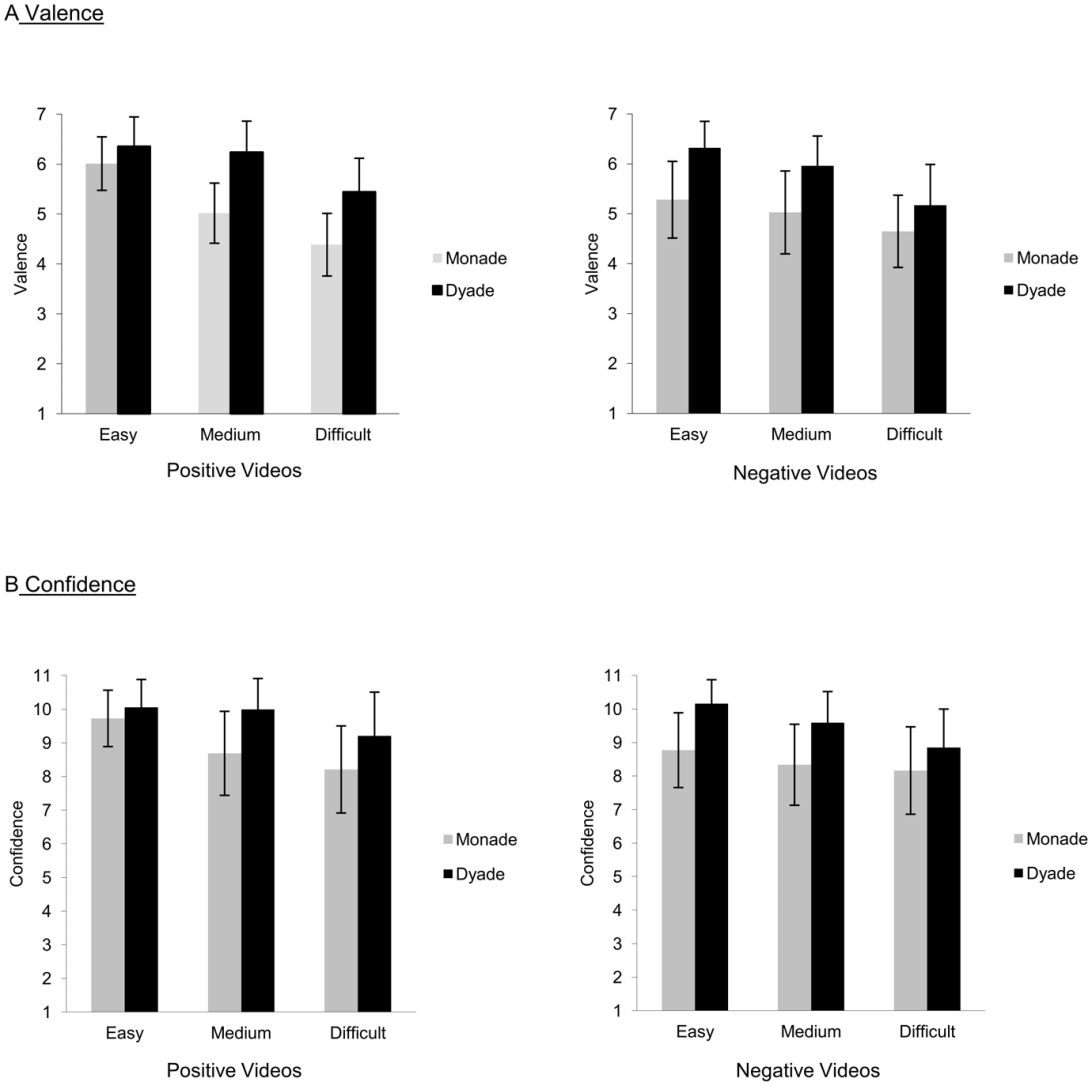
Data on the factors valence, social context and difficulty. Average rating data and standard errors for all levels of valence, social context (monadic and dyadic) and difficulty of stimuli for the valence rating (A) and the confidence rating (B).

The influence of the factors valence, social context and difficulty on the valence rating can best be understood in light of the significant three-way interaction (Figure 4, see Table 1 for statistical data on this section). When depicted valence was positive, differences between monades and dyades were smaller for easy videos compared to medium and difficult videos. When depicted valence was negative, the effect was reversed, namely, differences between monades and dyades were larger for easy videos compared to medium and difficult videos. Moreover, there was a significant main effect of the social context (monade vs. dyade) of the PLDs: PLDs depicting dyades were rated more positively or negatively than those depicting monads. There was also a significant main effect of difficulty: Easy PLDs were rated most positively or negatively; medium PLDs, less positively or negatively; and difficult PLDs, least positively or negatively (Figure 4A).

Also for the confidence rating a significant three-way interaction between valence, social context, and difficulty was found (Figure 4B): When depicted valence was positive, differences between monades and dyades were smaller for easy videos compared to medium and difficult videos. When depicted valence was negative, the effect was reversed, namely, differences between monades and dyades were larger for easy videos compared to medium and difficult videos. Moreover, there was a significant main effect of valence: Participants were more confident after evaluating videos with positive content than videos with negative content. There was also a significant main effect of social context: Participants were more confident about assessing dyades than monades. The main effect of difficulty was also significant: Easy PLDs were rated more confidently than medium and difficult PLDs.

## Discussion

The present study aimed to investigate the effect of different values of the trait alexithymia on perceiving emotions in PLDs of varying difficulty depicting either monades or dyades. More precisely, we tried to elucidate how the ability to recognize and describe one's own emotions relates to the perception of emotions depicted in PLDs.

On this background, the present data demonstrated that people with a higher ability to perceive and recognize own emotions (i.e., those with lower alexithymia scores) reported an increased confidence in their emotion perception, but did not differ in the rated valence, although, people with higher alexithymia scores demonstrated smaller differences between videos with varying difficulty. No modulating effect of social context on the effect of higher alexithymia scores was found.

Our findings, demonstrated, furthermore, the importance of contextual that is, social information for emotion perception per se. We found that perception of social interaction facilitates the perception of emotional states as well as how confident participants are about their evaluation of both positive and negative emotions in videos with varying difficulty—and this finding is independent from participants' alexithymia scores. This is in line with a study conducted by Clarke et al. [11] demonstrating that emotions displayed by dyades are perceived significantly better than those displayed by monades. The results indicate that humans use contextual social information when perceiving and recognizing emotions. As these findings were not relevant for the present main research question, the following sections will only discuss the findings on alexithymia and their implications in more detail.

### 4.1. The Effect of Alexithymia on Emotion Perception

People with alexithymia have trouble in identifying and describing their own feelings and emotions. Typical deficits may be found in identifying, describing, and working with own feelings [29], [30]. However, because we are social animals, the ability to perceive, recognize, and understand the emotions of our conspecifics is considered to be a cornerstone of human social life. On the social level, people with alexithymia demonstrate interpersonal problems, for example, they tend to avoid emotionally close relationships. Clinical observations report that people with alexithymia tend to be un-empathic, cold, and detached [31]. Chaotic relationships [32] as well as an inadequate differentiation between self and other have also been observed [33]. Empirical data have demonstrated that alexithymic persons describe themselves as distant and nonassertive in social relationships [31], and that alexithymia might be associated with an impaired understanding and demonstration of relational affection [34]. Furthermore, it was stated that alexithymic individuals demonstrate a lack in self-confidence [35], [36]. Regarding these deficits, several experimental approaches have demonstrated that people with alexithymia also lack understanding of the feelings of others [7], [8], [37]–[40]. In line with this notion, the major finding in this study is that it is particularly the confidence in rating emotions depicted in PLDs that decreases as alexithymia scores increase in a healthy population. This decreased confidence in rating emotional stimuli suggests that the perception of emotions of others might be influenced by the ability to recognize and evaluate one's own feelings and emotions. As the present data stem from a subclinical sample, one might speculate that in a healthy sample only the confidence is influenced by the factor alexithymia whereas in a patient sample also the valence detection in PLDs might be influenced (for example, [7], [8]). Support for this speculation is that patients with other neuropsychiatric disorders such as autism spectrum disorders, which are also characterized by social cognition problems, prove to have impairments in biological motion perception when observing PLDs [41]–[43].

The present study is the first to use PLDs of either one or two persons depicting emotional scenes to investigate the effect of alexithymia on emotion perception. These displays provided exclusively kinematic movement information. Hence, it might be speculated even the processing of emotional body information might be influenced by alexithymia, thus underpinning the often propagated notion that it is not just a problem in the use of words to describe emotions but rather a problem in processing emotions, emotional scenes, emotional faces, and even biological movement patterns depicting emotions [7], [8], [37]–[40]. Against this background, Prince and Berenbaum [44] have demonstrated that alexithymic individuals often experience little pleasure in social situations. One possible reason for this might be an increased uncertainty in perceiving, recognizing, and evaluating emotions in observed persons or even their interactions. Recognizing and putting oneself in the position of another person are important interpersonal abilities in social situations, and deficits in these abilities can lead to uncertainty in social relationships and a tendency toward isolation.

### 4.2. Deficits in the Embodied Simulation of Emotions?

One central argument in social cognitive neuroscience has been that the understanding of observed emotions might be based on an embodied simulation process [4], [21], [45]. In this regard, seeing someone else's emotional behavior might be linked to a simulation of the respective emotion and, therefore, to experiencing own emotions. On a neural level, Wicker et al. [21] have shown that observing another person's disgust results in activation of the insula—an area in the human brain activated when we experience disgust ourselves. In the present experiment, we demonstrated that people who have a shortcoming in experiencing own emotions were significantly more uncertain about evaluating observed emotional scenes depicted by PLDs, even though they did not make significantly more mistakes. One speculation is that using one's own representation to simulate observed emotions enhances certainty. Thus, persons with a shortcoming in perceiving, recognizing, and describing own emotions show the same performance when evaluating a scene, but, however, a reduced certainty about their evaluation because they may well be drawing on a more cognitive strategy rather than engaging in simulation [4], [46].

Further, alexithymic individuals were described as more uncertain in social situations [41] and they also lack in self-confidence [35], [36]. Thus, an alternative explanation for the present results might be that those characteristics influenced the confidence rating of the participants with a higher TAS-26-score. Both possible explanations would need to be confirmed with, for example, brain imaging methods or the usage of alternative questionnaires, before any final conclusion can be reached on this topic.

## Conclusions

First and foremost, we have demonstrated that people with a higher ability to perceive and recognize own emotions are more confident about assessing others' emotions. PLDs of monadic and dyadic emotion depiction addressed emotion perception without using verbal or facial information. The difference in the rated confidence might point to the notion that working with own feelings enhances certainty of emotion perception. The association of emotion understanding via kinematic information and the ability to perceive own emotions might be more pronounced in patient samples. Furthermore, the present data replicate the finding that perception of a social context enhances the perception of specific emotions as well as the participants' confidence in their evaluation of this process regardless of their individual alexithymia score. This holds for both positive and negative stimuli with varying difficulty. Thus, humans use contextual social information when perceiving emotions in abstract PLDs.
